# Multiple Aneurysms and Thrombotic Events as Initial Manifestations of Primary Myelofibrosis: A Case Report

**DOI:** 10.7759/cureus.79519

**Published:** 2025-02-23

**Authors:** Konstantinos Manganas, Thrasyvoulos Bemplidakis, Klairi Papachristou, Maria Angelara, George Karamanakos

**Affiliations:** 1 First Department of Propaedeutic and Internal Medicine, Laiko General Hospital, Athens, GRC

**Keywords:** aneurysms, deep venous thrombosis, jak2v617f, primary myelofibrosis, pulmonary embolism

## Abstract

This case report presents a 66-year-old male who developed deep venous thrombosis (DVT), pulmonary embolism (PE), and a ruptured iliac aneurysm as initial manifestations of primary myelofibrosis (PMF). Due to the presence of pre-existing aneurysms in combination with anticoagulation therapy, the patient experienced a retroperitoneal hematoma, necessitating temporary cessation of treatment. Genetic testing revealed a JAK2 V617F mutation and bone marrow biopsy confirmed PMF. The patient’s recovery was uneventful, with hematological parameters stabilized upon discharge. The case emphasizes the importance of considering myeloproliferative neoplasms (MPNs) in the differential diagnosis of unexplained thrombotic events. JAK2 mutations are linked to thrombotic complications and aneurysm formation, highlighting the need for vigilant monitoring. It is also important that MPNs may not initially be evident in a complete blood count, while coexisting conditions, such as β-thalassemia trait in this patient’s case, can alter the blood count findings.

## Introduction

Myeloproliferative neoplasms (MPNs) represent a group of hematologic disorders marked by the abnormal proliferation of blood cells in the bone marrow [1]. MPNs encompass three major disorders: polycythemia vera (PV), essential thrombocythemia (ET), and primary myelofibrosis (PMF). Unlike PV and ET, which are primarily characterized by erythrocytosis and thrombocytosis, respectively, PMF is distinguished by progressive bone marrow fibrosis, ineffective hematopoiesis, and extramedullary hematopoiesis, often leading to cytopenias and splenomegaly. These pathophysiological differences influence both clinical presentation and thrombotic risk, making PMF a unique diagnostic and therapeutic challenge. Thrombotic events, both arterial and venous, are a common but often underappreciated complication in PMF [2]. These findings are significant because thrombotic events, especially in the absence of traditional risk factors, should prompt consideration of underlying hematologic disorders, particularly in older patients or those with abnormal blood counts.

The presence of a JAK2 V617F mutation further solidifies the diagnosis of PMF, as this mutation is found in approximately 50-60% of PMF cases and is associated with a higher risk of thrombotic complications [3]. This mutation drives excessive blood cell production, increasing blood viscosity and predisposing patients to both clot formation and bleeding risks [4,5]. Additionally, JAK2 mutations are implicated in the formation of aneurysms, suggesting a potential link between MPNs and vascular abnormalities [6]. This case underscores the need for heightened clinical awareness of PMF and MPNs when patients present with thrombotic events that are unexplained, when they occur in the absence of traditional risk factors, or atypical, such as thrombosis in unusual locations or concurrent arterial and venous involvement. Early recognition of MPNs is critical, as timely diagnosis and management - such as the use of hydroxyurea, anticoagulation, or even targeted therapies - can mitigate complications, improve patient outcomes, and potentially prevent life-threatening events like massive thromboses or aneurysmal ruptures. Furthermore, this case illustrates the challenge of balancing anticoagulation therapy in MPN patients, given the simultaneous risk of thrombotic and hemorrhagic events.

## Case presentation

**Figure 1:**
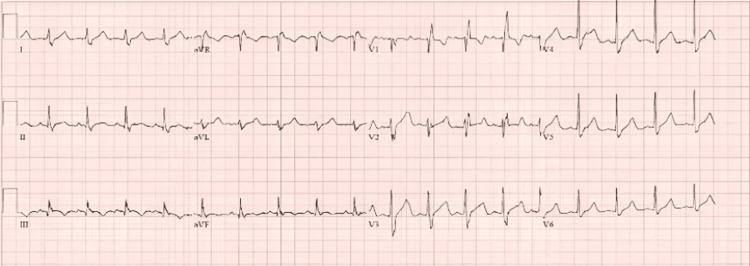
Patient’s electrocardiogram (ECG) showing S1Q3T3 pattern characteristic of pulmonary embolism.

This case report details the clinical presentation and management of a 66-year-old male with no significant past medical history apart from β-thalassemia trait, who was diagnosed with pulmonary embolism (PE) and multiple aneurysms as first manifestations of primary meylofibrosis. The patient initially presented to the emergency department of "Laiko" General Hospital in Athens, Greece, following a brief episode of loss of consciousness while seated, with no preceding or subsequent symptoms. He reported no smoking or illicit drug use. The patient presented with left lower limb deep venous thrombosis (DVT), evidenced by swelling, redness, and tenderness. Notably, his chest radiograph was normal, but an electrocardiogram revealed sinus tachycardia with an S1Q3T3 pattern (Figure 1).

Laboratory findings indicated a hemoglobin level of 15.7 g/dl, high RBC count, elevated troponin and D-dimer levels, with other blood parameters within acceptable ranges. The patient’s initial laboratory values are summarized in Table 1.

**Table 1:** Patient’s initial laboratory values during admission.

Parameter	Patient Values	Reference Range
White Blood Cells (WBC)	7.05 Κ/μl	4.5-11 K/μl
Red Blood Cells (RBC)	6.64 M/μl	4.6-6.2 M/μl
Hematocrit (Hct)	50%	40-54
Reticulocytes	0.139 Μ/μl	0.02-0.160 Μ/μl
Hemoglobin (Hb)	15.7 g/dl	13.5-18 g/dl
Mean Corpuscular Volume (MCV)	75.6 fl	80-96 fl
Mean Corpuscular Hemoglobin (MCH)	24.1 fl	27-31 fl
Platelets	372.000 K/μl	140-440 K/μl
Troponin	40 pg/ml	<14 pg/ml
NT-proBNP	2168 pg/ml	<349 pg/ml
D-dimers	7.03 μg/ml	<0.5 μg/ml
Erythrocyte Sedimentation Rate	30 mm/hr	0-20 mm/hr
Glucose	80 mg/dl	72-106 mg/dl
Urea	71 mg/dl	15-43 mg/dl
Creatinine	1.10 mg/dl	0.7-1.2 mg/dl
Sodium	137 mmol/l	136-143 mmol/l
Potassium	4.9 mmol/l	3.7-4.9 mmol/l
Aspartate transferase (AST)	28 U/L	15-40 U/L
Alanine Transaminase (ALT)	14 U/L	<41 U/L
Lactate dehydrogenase (LDH)	306 U/L	135-225 U/L
Creatine phosphokinase (CPK)	79 U/L	38-190 U/L
C-Reactive Protein	30 mg/L	<0.5 mg/L

**Figure 2:**
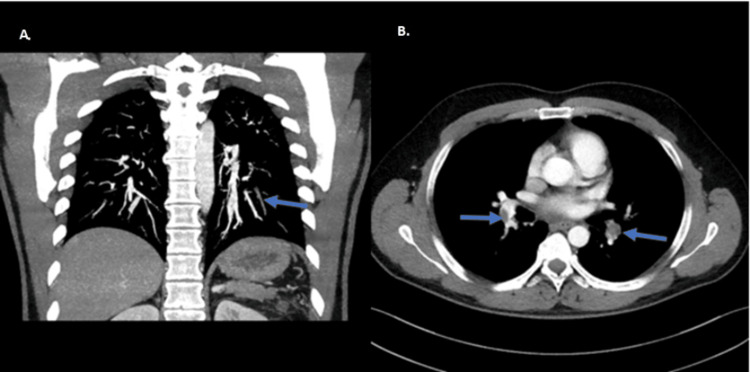
Computed tomography pulmonary angiography revealing unilateral subsegmental pulmonary embolism (blue arrows). A: coronal plane and B: axial plane

A computed tomography pulmonary angiography (CTPA) confirmed the presence of filling defects in segmental and subsegmental branches bilaterally, revealed an right ventricle to left ventricle (RV/LV) ratio of 1 and showed aneurysmal dilation of the thoracic aorta (4.2 cm) (Figure 2).

A cardiac ultrasound demonstrated a strain pattern of the right ventricle with RV systolic pressure (RVSP) of 46 mmHg, suggesting mild pulmonary hypertension, and flattening of the interventricular septum due to pressure and volume overload of the right ventricle, with a pulmonary artery systolic pressure (PASP) of 45mmHg. Additionally, a lower limb triplex examination revealed thrombosis in the left superficial and common femoral vein and the corresponding popliteal vein, aneurysmal dilation of the popliteal arteries bilaterally, with the presence of a thrombus in the right popliteal artery exhibiting biphasic flow. The arterial ultrasound was performed due to the incidental finding of popliteal artery aneurysms during venous ultrasound, necessitating further evaluation of the arterial circulation, even though this is not standard practice. The right leg was also included in the scan based on clinical suspicion, to rule out bilateral deep venous thrombosis, despite the patient not exhibiting signs of DVT in the right leg.

**Figure 3:**
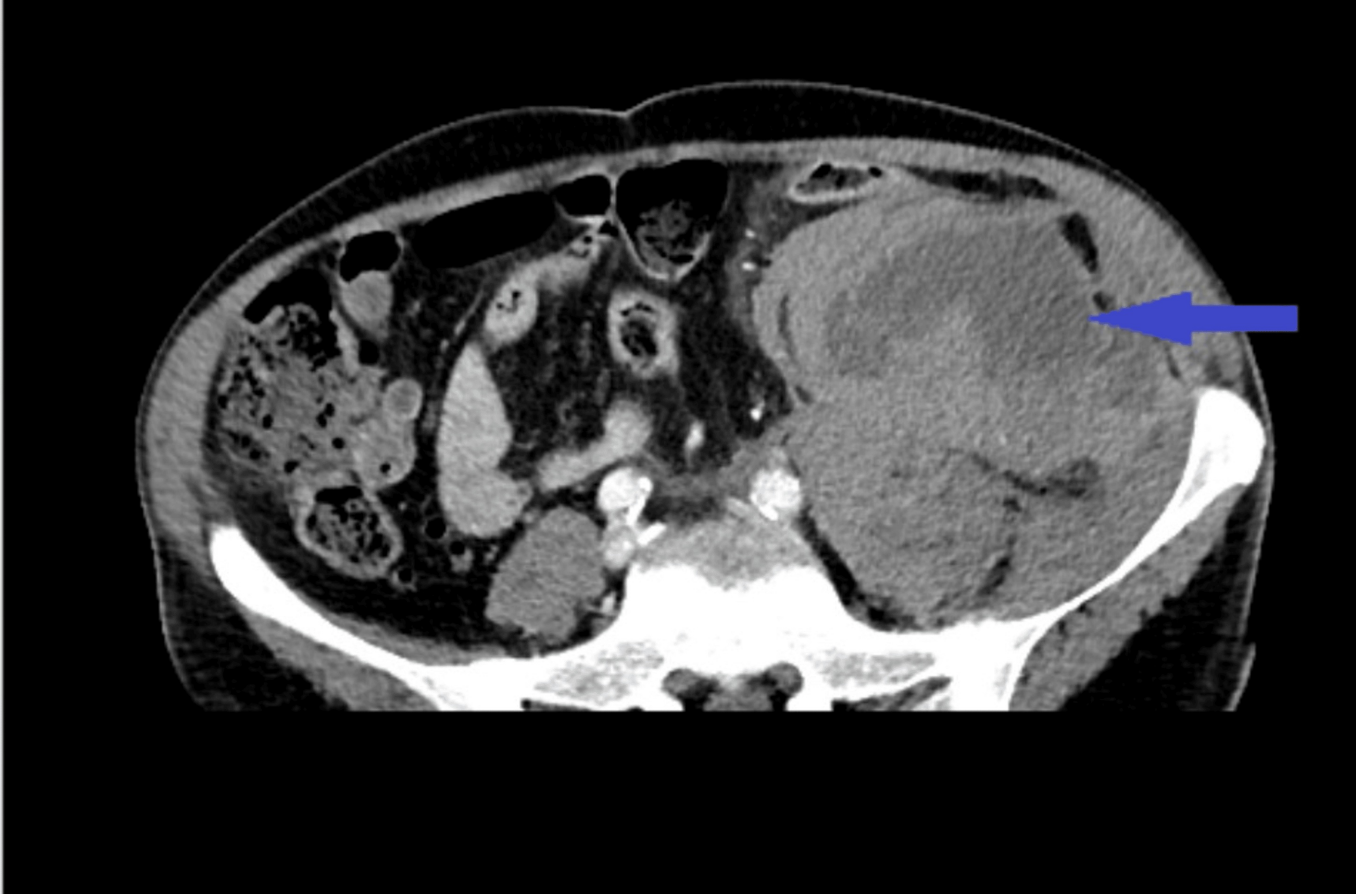
CT angiography of the patient revealing an actively bleeding large hematoma 25 x 9 cm in the retroperitoneal cavity (blue arrow).

According to the Pulmonary Embolism Severity Index (PESI) criteria and risk stratification for pulmonary embolism, the patient was placed in the intermediate-high risk group, due to high troponin and N-terminal pro-brain natriuretic peptide (NT-proBNP) levels and right ventricular dysfunction, without hemodynamical instability. Prompt interventions were initiated, including oxygen therapy and anticoagulation with low molecular weight heparin (LMWH). However, on the second day of hospitalization, while on enoxaparin therapy from admission, the patient developed left abdominal pain with tenderness, accompanied by a significant drop in hemoglobin levels. Subsequent CT angiography revealed an actively bleeding large hematoma measuring 25x9 cm in the retroperitoneal cavity (Figure 3).

Managing a patient with active bleeding and concomitant pulmonary embolism was particularly challenging. Anticoagulation therapy was immediately discontinued. Invasive angiography further revealed multiple aneurysms in the abdominal aorta and common iliac arteries, along with a bleeding site from a ruptured aneurysm of the left iliac artery, which was successfully treated with endovascular repair. With a new imaging study, one day after invasive angiography, we confirmed there was no active bleeding. Placing an inferior vena cava filter was considered for secondary prophylaxis, but it was technically not possible due to anatomical variation in the left renal vein. Anticoagulation therapy with enoxaparin was gradually re-initiated after five days from discontinuation, while monitoring anti-Xa levels. The patient did not experience new bleeding or a drop in hemoglobin during hospitalization, and there was improvement in oxygenation parameters, allowing discontinuation of oxygen therapy.

**Figure 4:**
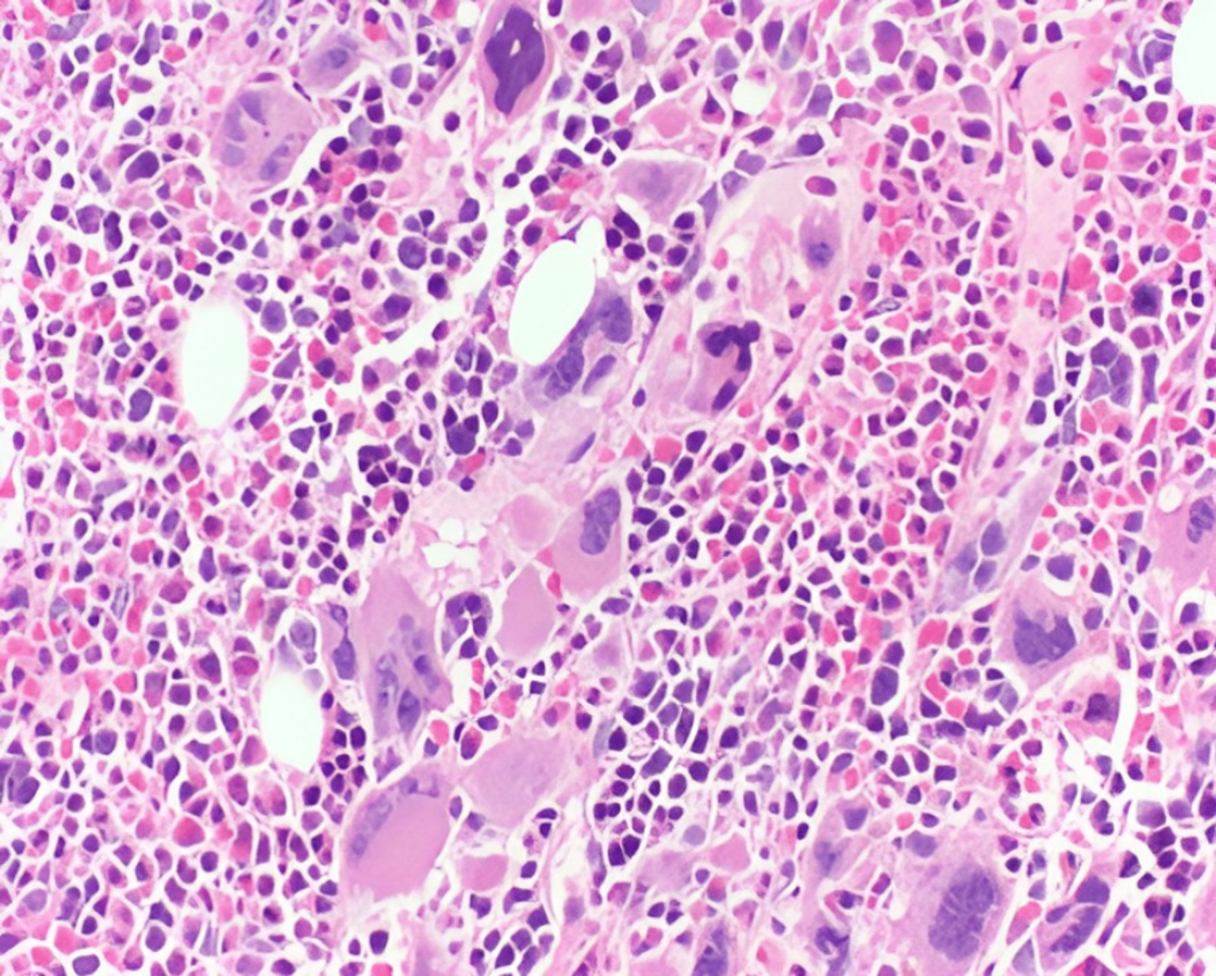
The bone marrow showing increased cellularity with megakaryocytic hyperplasia (hematoxylin and eosin stain, ×200)

Thorough investigation for deep venous thrombosis and pulmonary embolism was conducted, encompassing thrombophilia testing for various genetic mutations such as MTHFR, protein C/S, factor V Leiden, and prothrombin 20210A. The results, however, were negative. Additional laboratory tests, including erythropoietin, C3, C4, rheumatoid factor, and homocysteine levels, were within normal limits, while antinuclear antibodies (ANA), β2-GPI, and anti-cardiolipin antibodies were negative. We observed a discrepancy in the patient's admission data, noting initially elevated red blood cell count and normal hemoglobin and hematocrit levels, which contradicted the reported history of heterozygous β-thalassemia. The peripheral blood smear revealed hypochromia and microcytosis, without any atypical findings. To validate the heterozygosity for β-thalassemia, we conducted hemoglobin electrophoresis and began considering the possibility of a myeloproliferative syndrome. Genetic testing yielded negative results for the BCR-ABL mutation but identified a positive JAK2 V617F mutation. Notably, bone marrow aspiration was unsuccessful (dry tap), but the subsequent biopsy indicated hypercellularity with increased megakaryocytes, patchy hematopoietic cellularity, and reticular fibrosis, indicative of primary myelofibrosis (Figure 4). 

The patient was subsequently treated with oral anticoagulation with apixaban, acetylsalicylic acid and hydroxyurea and discharged from the hospital after a 15-day stay. Follow-up instructions were given for outpatient reassessment. He discontinued oral anticoagulation after a total of three months of therapy and serial D-dimer measurements remained below the threshold. In repeat imaging studies after a few months, the retroperitoneal hematoma was absorbed, and the patient remains asymptomatic with good hematological parameters.

## Discussion

This case report highlights the atypical presentation of PMF with DVT, PE, and a ruptured iliac aneurysm as the initial manifestations. Thrombotic events, both arterial and venous, are known to be associated with MPNs, and this case emphasizes the importance of considering MPNs in the differential diagnosis of unexplained thrombotic events. In PMF, the incidence rate of major thrombotic events is approximately 2.2% per patient-year. This rate is comparable to that observed in ET and highlights the significant risk of thrombosis in PMF patients, particularly those with the JAK2 V617F mutation and leukocytosis [7]. Elliot et al. reported an incidence of thrombotic events of 13.2% at or prior to diagnosis, and 10.7% over a median follow-up of 31 months in a series of 208 patients [8].

In this case, the patient's initial presentation with loss of consciousness and subsequent findings of DVT and PE prompted a thorough investigation to identify the underlying cause. Thrombophilia testing is generally not recommended for an unprovoked first episode of venous thromboembolism (VTE) or pulmonary embolism [9]. However, in this case, the patient’s atypical presentation, characterized by concurrent arterial and venous thromboses, warranted a comprehensive thrombophilia workup to investigate a possible underlying prothrombotic disorder. Despite having a history of heterozygous β-thalassemia, this patient presented with notably elevated levels of red blood cells and normal hematocrit and hemoglobin levels, which is a significant finding and further guides the diagnosis of a hematological disorder and this was the key to our diagnosis. Moreover, MPNs may not initially be evident in a complete blood count. Coexisting conditions, such as β-thalassemia trait in this patient’s case, even though they are not related with vascular complications can alter the blood count findings and lead to a delay in diagnosis [10].

The presence of the JAK2 V617F mutation, commonly found in MPNs, further supports the diagnosis of PMF in this patient, as JAK mutations are present in over half the cases of PMF [11]. In addition to causing hemorrhagic and thrombotic complications, JAK2 mutations are also associated with an increased risk of cardiovascular disease [12]. Interestingly, recent literature has also associated JAK2 V617F mutation with the development and rupture of aortic aneurysms [6]. The underlying mechanism possibly involves JAK2 V617-positive circulating leukocytes that demonstrate upregulation of genes associated with aortic aneurysm formation, including matrix metalloproteinase 9 (MMP-9) [13]. In this case, the subsequent discovery of multiple aneurysms in the thoracic aorta, abdominal aorta and common iliac arteries highlight the potential association between JAK2 V617F mutation and aneurysm formation. Although the association between JAK2 V617F mutation and vascular abnormalities, including aneurysm formation, has been increasingly recognized, routine aneurysm screening is not currently recommended for MPN patients with JAK2 mutations, as evidence remains limited and no consensus guidelines exist.

Management of this patient involved a multidisciplinary approach. Prompt initiation of oxygen therapy and anticoagulation with LMWH was crucial in the management of acute pulmonary embolism. The subsequent development of abdominal pain and drop in hemoglobin levels indicated active bleeding and necessitated further evaluation and intervention. Endovascular repair of multiple aneurysms was performed to address the patient's abdominal hemorrhage. Management of anticoagulation therapy in a patient with pulmonary embolism and active bleeding is particularly challenging, as there are no clear guidelines on the discontinuation and resumption of anticoagulants, and therapeutic decisions must be individualized. On the topic of anticoagulant-related bleeding, the most extensively studied are gastrointestinal hemorrhages. A systematic review found that resuming oral anticoagulation after gastrointestinal bleeding lowers thromboembolism risk and death, but increases recurrent bleeding risk. Studies suggest restarting oral anticoagulation at discharge or more than seven days post-bleed improves outcomes. It's generally reasonable to restart oral anticoagulation once hemostasis is achieved [14]. In our clinical case, due to the patient's high risk of complications from a recent pulmonary embolism and the successful achievement of hemostasis through embolization, we decided to restart anticoagulation earlier, at lower doses, while monitoring anti-Xa levels. Enoxaparin (LMWH) was chosen as a bridge in this patient due to prior hemorrhagic complications, as LMWH have a shorter half-life and their action can be monitored with anti-Xa levels, which made LMWH a safer option at the time.

Vitamin K antagonists (VKAs) are the historic anticoagulant recommended for use in MPNs. Direct oral anticoagulants (DOACs) appear to have a similar risk-benefit profile to VKAs in MPN patients and could represent a possible alternative to VKAs, although clear anticoagulation guidelines for MPN-related thrombosis and prevention are lacking and evidence of DOACs efficacy and safety in MPN is very limited and based on few retrospective observational studies. Studies report a notable bleeding risk, particularly in PMF patients and those receiving dabigatran, highlighting the need for careful patient selection [15]. The decision to discontinue oral anticoagulation after three months of treatment was initially based on the patient’s preference. The patient was closely monitored with serial D-dimer measurements, which remained below the threshold after discontinuation in serial measurements, indicating a lower risk of recurrence [16]. 

## Conclusions

This case report highlights the importance of recognizing MPNs, particularly PMF, as a possible cause of unexplained thrombotic events. Unexplained or atypical thrombotic events, particularly in the absence of major cardiovascular risk factors, should prompt evaluation for MPNs, even if CBC findings appear normal, as MPNs may not present with obvious hematological abnormalities in early stages, making genetic testing and further investigations crucial. The presence of the JAK2 V617F mutation strengthens the suspicion of an MPN and may indicate a higher risk of both thrombosis and aneurysm formation. While JAK2 mutations may contribute to a pro-inflammatory and pro-thrombotic environment, which could theoretically influence vascular integrity, direct evidence of their role in aneurysm formation is still limited. A deeper understanding of the connection between MPNs, thrombosis, and vascular abnormalities may help refine future diagnostic and treatment approaches.

## References

[1] Tremblay D, Yacoub A, Hoffman R (2021). Overview of myeloproliferative neoplasms: history, pathogenesis, diagnostic criteria, and complications. Hematol Oncol Clin North Am.

[2] Kc D, Falchi L, Verstovsek S (2017). The underappreciated risk of thrombosis and bleeding in patients with myelofibrosis: a review. Ann Hematol.

[3] Barbui T, Finazzi G, Falanga A (2013). Myeloproliferative neoplasms and thrombosis. Blood.

[4] Moliterno AR, Kaizer H, Reeves BN (2023). JAK2 V617F allele burden in polycythemia vera: burden of proof. Blood.

[5] Fleischman AG, Tyner JW (2013). Causal role for JAK2 V617F in thrombosis. Blood.

[6] Yokokawa T, Misaka T, Kimishima Y (2021). Crucial role of hematopoietic JAK2 V617F in the development of aortic aneurysms. Haematologica.

[7] Barbui T, Carobbio A, Cervantes F (2010). Thrombosis in primary myelofibrosis: incidence and risk factors. Blood.

[8] Elliott MA, Pardanani A, Lasho TL, Schwager SM, Tefferi A (2010). Thrombosis in myelofibrosis: prior thrombosis is the only predictive factor and most venous events are provoked. Haematologica.

[9] Ortel TL, Neumann I, Ageno W (2020). American Society of Hematology 2020 guidelines for management of venous thromboembolism: treatment of deep vein thrombosis and pulmonary embolism. Blood Adv.

[10] Büyükaşık E, Güvenç B (2024). MPN-233 the intersection of thalassemia minor and chronic myeloproliferative disorders: clinical and laboratory implications. Clin Lymphoma Myeloma Leuk.

[11] Luque Paz D, Kralovics R, Skoda RC (2023). Genetic basis and molecular profiling in myeloproliferative neoplasms. Blood.

[12] Leiva O, Hobbs G, Ravid K, Libby P (2022). Cardiovascular disease in myeloproliferative neoplasms: JACC: CardioOncology state-of-the-art review. JACC CardioOncol.

[13] Elf SE (2021). JAK out of the box: myeloproliferative neoplasms--associated JAK2 V617F mutations contribute to aortic aneurysms. Haematologica.

[14] Tomaselli GF, Mahaffey KW, Cuker A (2020). 2020 ACC expert consensus decision pathway on management of bleeding in patients on oral anticoagulants: a report of the American College of Cardiology Solution Set Oversight Committee. J Am Coll Cardiol.

[15] Barbui T, De Stefano V, Carobbio A (2021). Direct oral anticoagulants for myeloproliferative neoplasms: results from an international study on 442 patients. Leukemia.

[16] Palareti G, Cosmi B, Legnani C (2014). D-dimer to guide the duration of anticoagulation in patients with venous thromboembolism: a management study. Blood.

